# Supplementary material for: Synergized regulation of NK cell education by NKG2A and specific Ly49 family members
Source: Nat Commun. 2019 Nov 1;10:5010. doi: 10.1038/s41467-019-13032-5 (PMC6825122; doi:10.1038/s41467-019-13032-5)
Supplement: Supplementary file 1 — Supplementary Information [file 41467_2019_13032_MOESM1_ESM.pdf]

Supplementary Information

Synergized regulation of NK cell education by NKG2A and specific Ly49 family members

Zhang et al.

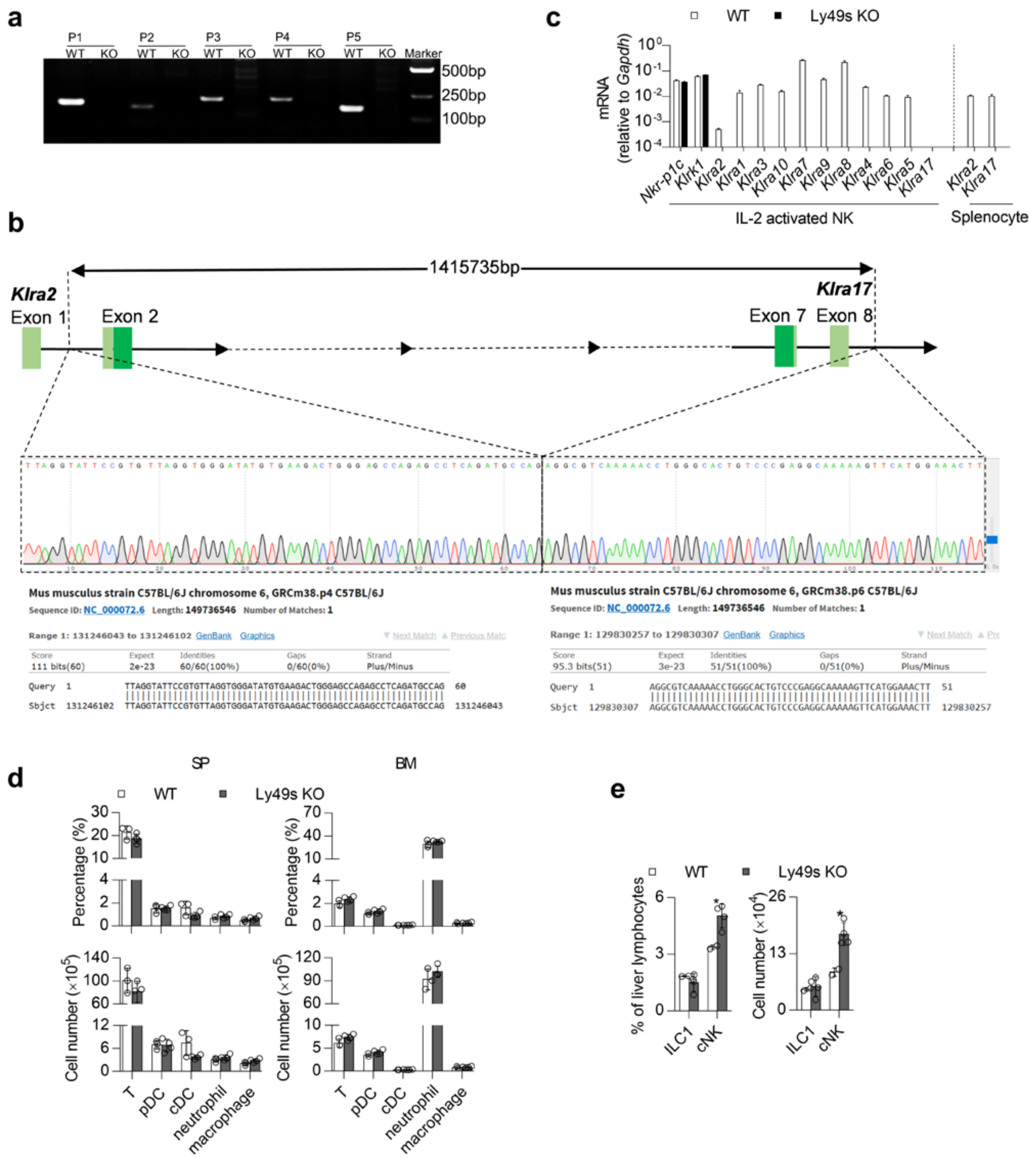

Supplementary Figure 1 | Generation and identification of Ly49-family deficient mice. a. Genomic PCR screening. P1 to P5 represent the five pairs of primers that are distributed in Ly49 sub-locus. Templates were genomic DNA obtained from the tail clippings of wild-type (WT) and mutant (KO) mice. **b.** Diagram (upper panel) of genome deletion of Ly49 locus from Ly49s KO mice. Light green boxes denote untranslated regions (UTR) and dark green boxes denote coding sequences (CDS) of mRNA of indicated genes. Partial display of genome sequencing results (middle panel). Dashed line gated regions are corresponding to the genome above. NCBI BLAST results of dashed line gated regions (lower panel). **c.** Total RNA was isolated from IL-2-expanded NK cells (left panel) or total splenocytes (right panel) from WT and Ly49s KO mice. Indicated genes were analyzed by quantitative

reverse transcription-PCR (RT-PCR). Data are shown as ratio of the indicated genes to *Gapdh*. **d.** Percentages and absolute numbers of splenic and bone marrow T cell ($CD3^{+}$NKR-$P1C^{-}$), pDC ($CD3^{-}$NKR-$P1C^{-}B220^{+}CD11c^{+}$), cDC ($CD3^{-}$NKR-$P1C^{-}B220^{-}CD11C^{+}$), neutrophil ($CD11b^{+}$Gr-1^{+}), macrophage ($CD11b^{+}F4/80^{+}$) from WT and Ly49s KO mice. **e.** Percentages and absolute numbers of liver ILC1 ($CD3^{-}NKp46^{+}CD49b^{-}CD49a^{+}$) and conventional NK ($CD3^{-}NKp46^{+}CD49b^{+}CD49a^{-}$) from WT and Ly49s KO mice. Each symbol represents an individual mouse. Data shown represent two (**c, d**) or at least three (**e**) independent experiments. Mean ± SD is shown. *$p < 0.05$. Unpaired Student's *t*-tests (two-tailed) was used to calculate these values. For the gate strategy of flow cytometry, refer to Supplementary Figure 9. Source data are provided as a Source Data file.

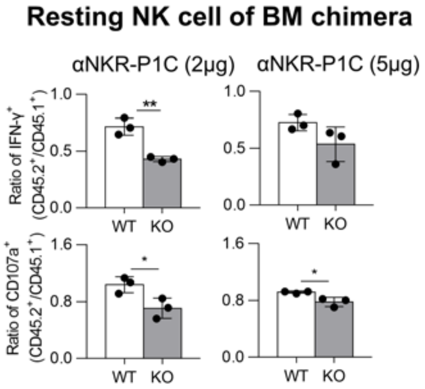

Supplementary Figure 2 | Ly49 family receptors intrinsically regulate NK cell responsiveness in bone marrow chimera assay. Naive splenocytes from BM chimeric mice (depicted in Fig. 1h) were stimulated with two doses (2 μg/ml and 5 μg/ml) of plate-bound anti-NKR-P1C. The relative ratio represents the percentage of IFN-γ^+ (upper panel) or CD107a^+ (lower panel) cells among gated CD45.2^+ NK cells compared to gated CD45.1^+ NK cells from the same chimeric mice. Each symbol represents an individual mouse. Data shown represent two independent experiments. Mean ± SD is shown. *$p < 0.05$, **$p < 0.01$, ***$p < 0.001$ and ****$p < 0.0001$. Unpaired Student’s *t*-tests (two-tailed) was used to calculate these values. Source data are provided as a Source Data file.

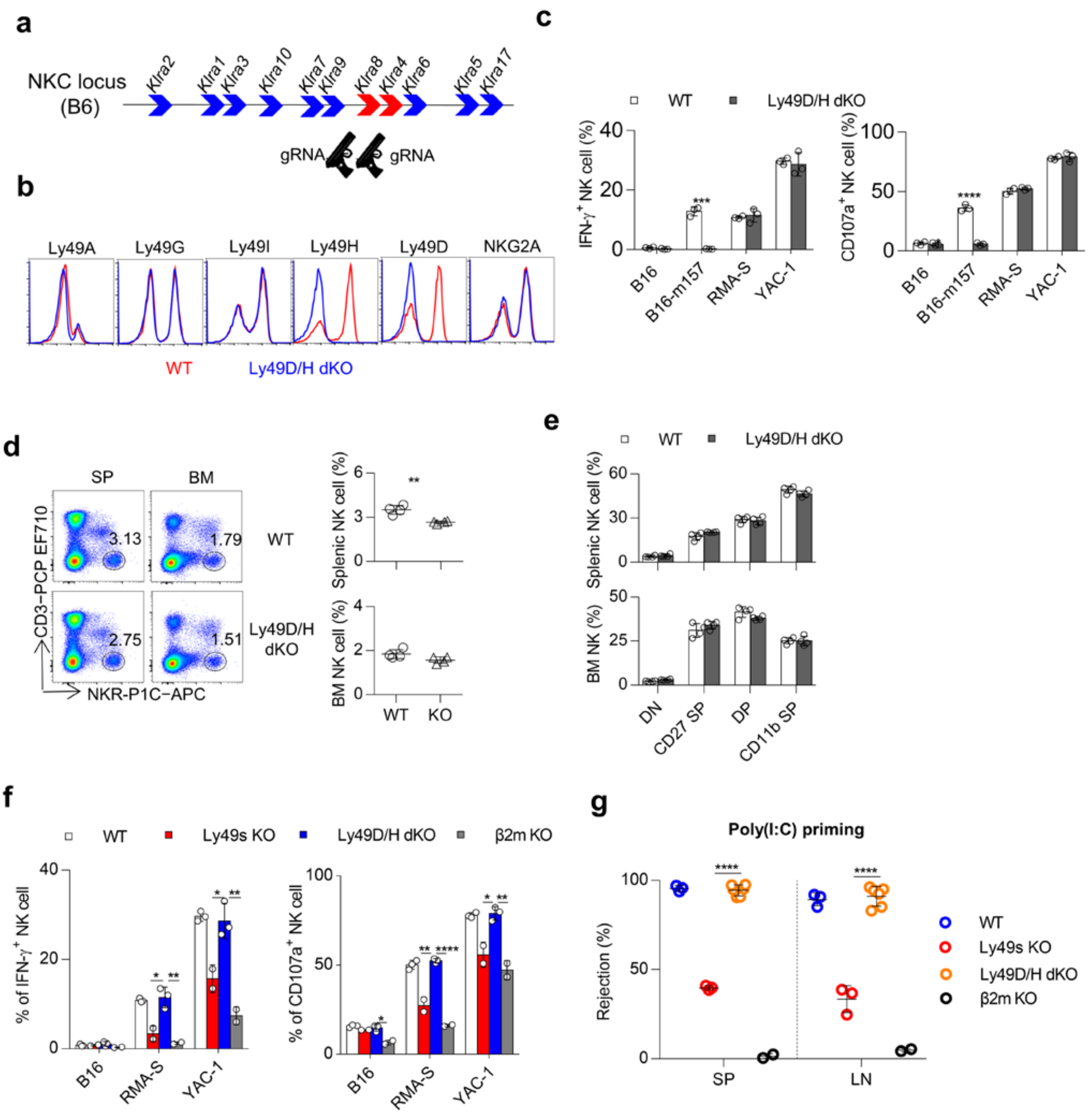

Supplementary Figure 3 | Deletion of Ly49D and H does not affect NK cell responsiveness. a. Diagram of Ly49-family genes in the NKC locus. Blue filled arrows denote inhibitory Ly49 receptors and red filled arrows denote activating Ly49 receptors. Guns denote CRISPR gRNAs targeting *Klra4* and *Klra8*. **b.** Flow cytometry analysis of the expression of the indicated receptors on splenic CD3$^-$NKp46$^+$ NK cells from WT (red line) and Ly49D/H dKO (blue line) mice. **c.** Poly(I:C)-primed splenocytes from the indicated mice were stimulated with tumour target cells. Percentages of IFN-γ^+ (left panel) and CD107a$^+$ (right panel) gated CD3$^-$NKp46$^+$ NK cells were analyzed. **d.** Representative flow cytometry plots (left panel) and percentages (right panel) of gated CD3$^-$NKR-P1C$^+$ NK cells in the spleen (SP) and bone marrow (BM) of WT and Ly49D/H dKO mice. **e.** Analysis of NK cell development. Percentages of gated CD3$^-$NKR-P1C$^+$ NK cells in the four stages of development, including DN (CD27$^-$CD11b$^-$), CD27 SP (CD27$^+$CD11b$^-$), DP (CD27$^+$CD11b$^+$), CD11b SP (CD27$^-$CD11b$^+$), in the spleen (upper panel) and bone marrow (lower panel) from WT and Ly49D/H

dKO mice. **f.** Poly(I:C)-primed splenocytes from WT and Ly49D/H dKO mice were stimulated by the indicated tumor targets. Frequency of IFN-γ^+ (left panel) and CD107a$^+$ (right panel) of gated splenic NK cells (CD3$^-$NKp46$^+$) were analyzed. **g.** In vivo rejection of β2M-deficient splenocytes (similar to **Fig. 3a**). Each symbol represents an individual mouse. Data shown represent two (**c-e**) or three (**f, g**) independent experiments. Mean ± SD is shown. *$p < 0.05$, **$p < 0.01$, ***$p < 0.001$ and ****$p < 0.0001$. Unpaired Student's *t*-tests (two-tailed) was used to calculate these values. Source data are provided as a Source Data file.

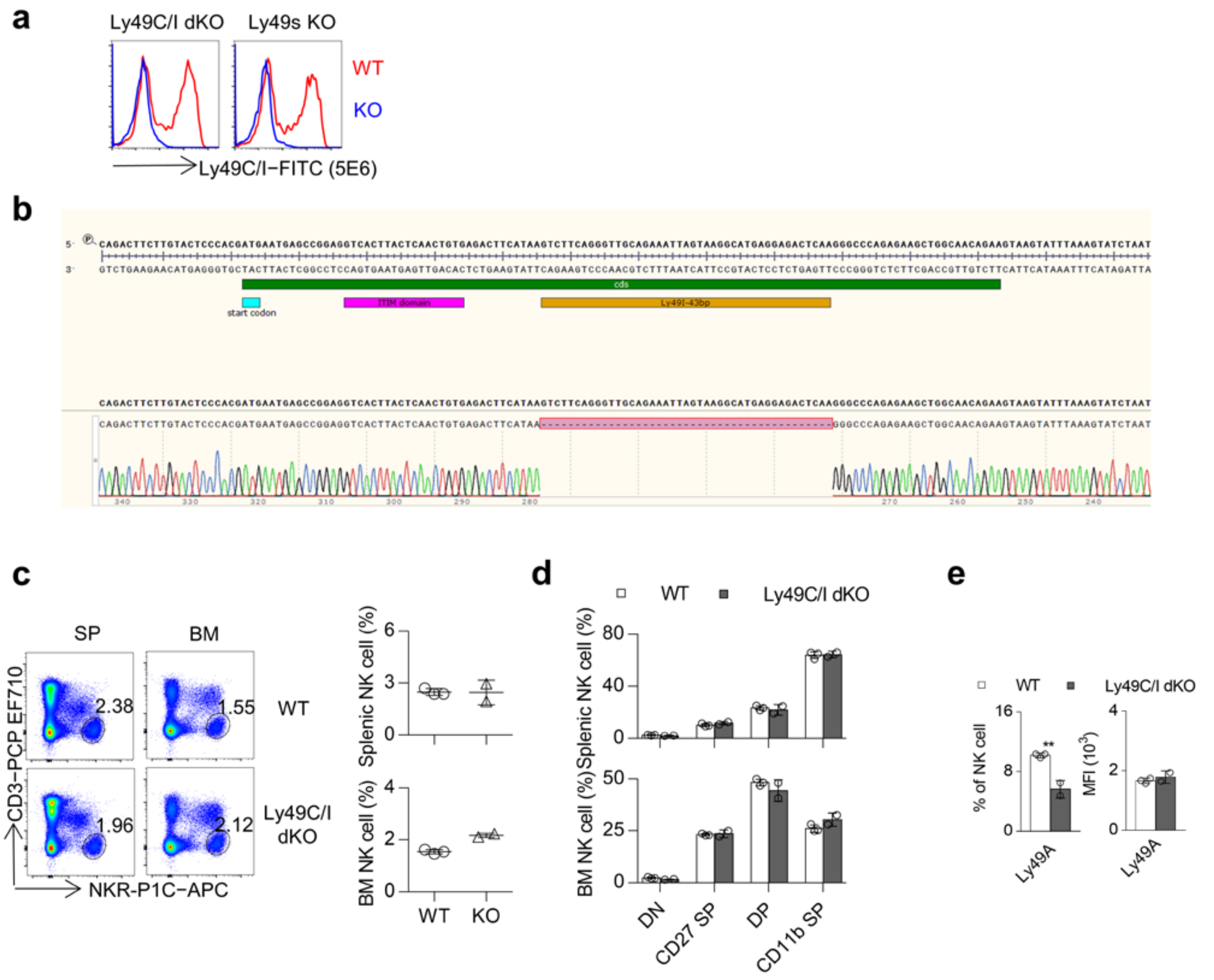

Supplementary Figure 4 | NK cell development in Ly49C/I dKO mice. a. Flow cytometry analysis of the expression of Ly49C and Ly49I on splenic NK cells (gated $CD3^{-}NKR\text{-}P1C^{+}$) from WT (red line) and indicated mutant (blue line) mice using 5E6 antibody. **b.** Sequencing of genome DNA of *Klra9* from Ly49C/I dKO mice (partial display). **c.** Representative flow cytometry plots (left panel) and percentages (right panel) of gated $CD3^{-}NKR\text{-}P1C^{+}$ NK cells in the spleen (SP) and bone marrow (BM) of WT and Ly49C/I dKO mice. **d.** Analysis of NK cell development. Percentages of gated $CD3^{-}NKR\text{-}P1C^{+}$ NK cells in the four stages of development, including DN ($CD27^{-}CD11b^{-}$), CD27 SP ($CD27^{+}CD11b^{-}$), DP ($CD27^{+}CD11b^{+}$), CD11b SP ($CD27^{-}CD11b^{+}$), in the spleen (upper panel) and bone marrow (lower panel) from WT and Ly49C/I dKO mice. **e.** The percentage and MFI of the Ly49A on splenic $CD3^{-}NKp46^{+}$ NK cells from WT and Ly49C/I dKO mice. Each symbol represents an individual mouse. Data shown represent two (**c-e**) independent experiments. Mean ± SD is shown. $**p < 0.01$. Unpaired Student's *t*-tests (two-tailed) was used to calculate these values. Source data are provided as a Source Data file.

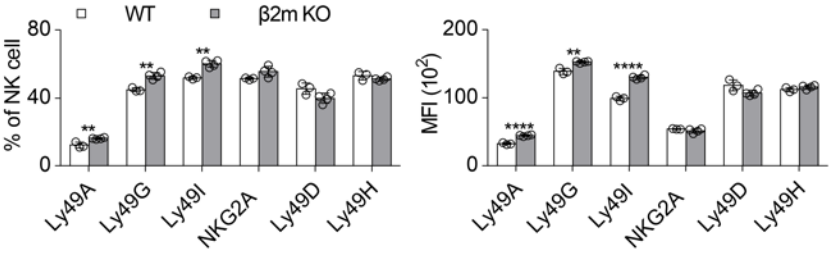

Supplementary Figure 5 | The expression of Ly49-family receptors in β2m-deficient mice. a. The percentages and MFIs of the indicated receptors on splenic $CD3^{-}NKp46^{+}$ NK cells from WT and $\beta 2m^{-/-}$ mice. Each symbol represents an individual mouse. Data shown represent two independent experiments. Mean ± SD is shown. $^{**}p < 0.01$ and $^{****}p < 0.0001$. Unpaired Student's *t*-tests (two-tailed) was used to calculate these values. Source data are provided as a Source Data file.

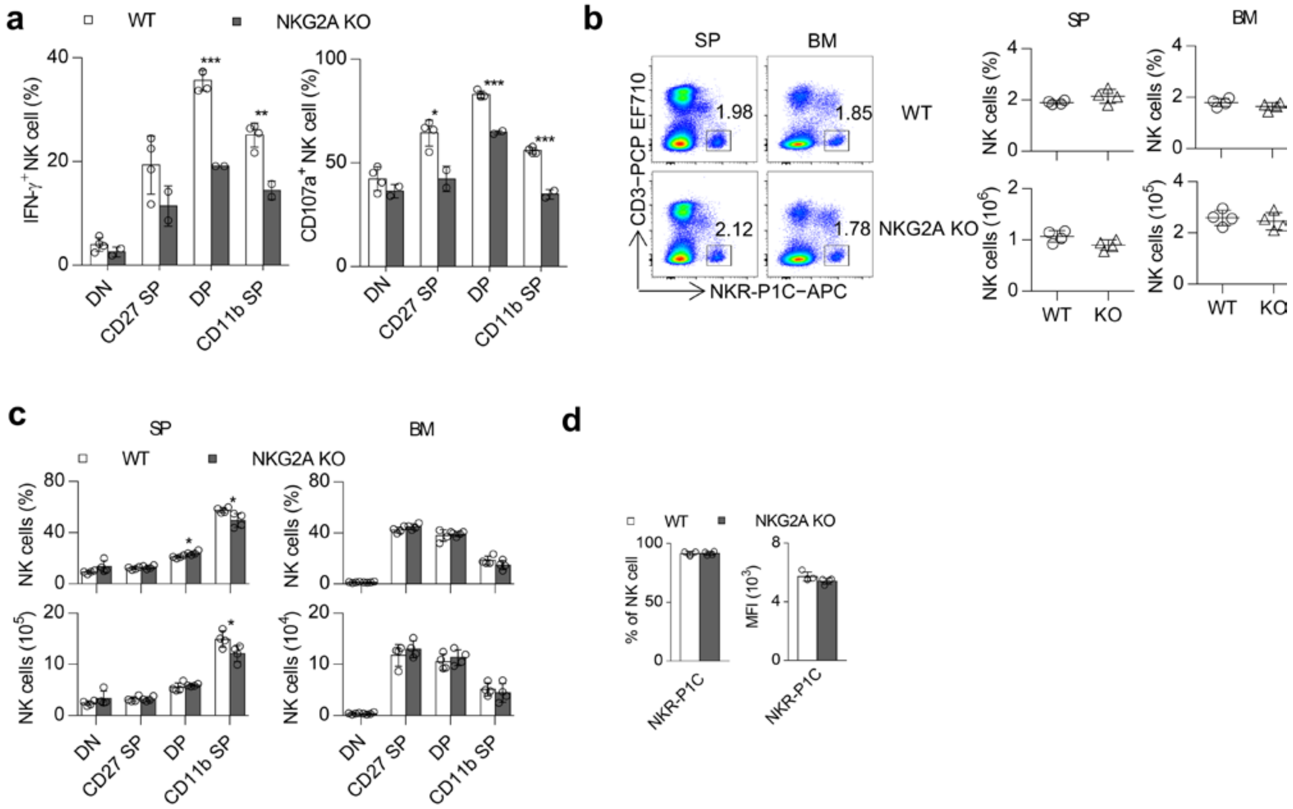

Supplementary Figure 6 | NK cell development in NKG2A-deficient mice. a. Poly (I:C)-primed splenocytes from WT and NKG2A-deficient mice (shown as NKG2A KO mice) were stimulated by YAC-1 tumor targets. Frequency of IFN-γ^+ (left panel) and CD107a^+ (right panel) of four-stage development, including DN ($CD27^-CD11b^-$), CD27 SP ($CD27^+CD11b^-$), DP ($CD27^+CD11b^+$), CD11b SP ($CD27^-CD11b^+$) of gated NK cells ($CD3^-NKp46^+$) were analyzed. **b.** Representative flow cytometry plots (left panel), percentages and absolute numbers of gated $CD3^-$NKR-P1C^+ NK cells in the spleen (middle panel) and bone marrow (right panel) of WT and NKG2A KO mice. **c.** Analysis of NK cell development. Percentages (upper panel) and absolute numbers (lower panel) of gated $CD3^-$NKR-P1C^+ NK cells in the four stages of development, including DN ($CD27^-CD11b^-$), CD27 SP ($CD27^+CD11b^-$), DP ($CD27^+CD11b^+$), CD11b SP ($CD27^-CD11b^+$), in the spleen and bone marrow from WT and NKG2A KO mice. **d.** The percentage and MFI of NKR-P1C in gated splenic $CD3^-CD122^+$ NK precursor cells from WT and NKG2A KO mice. Each symbol represents an individual mouse. All data shown represent two independent experiments. Mean ± SD is shown. $*p < 0.05$, $**p < 0.01$ and $***p < 0.001$. Unpaired Student's *t*-tests (two-tailed) was used to calculate these values. Source data are provided as a Source Data file.

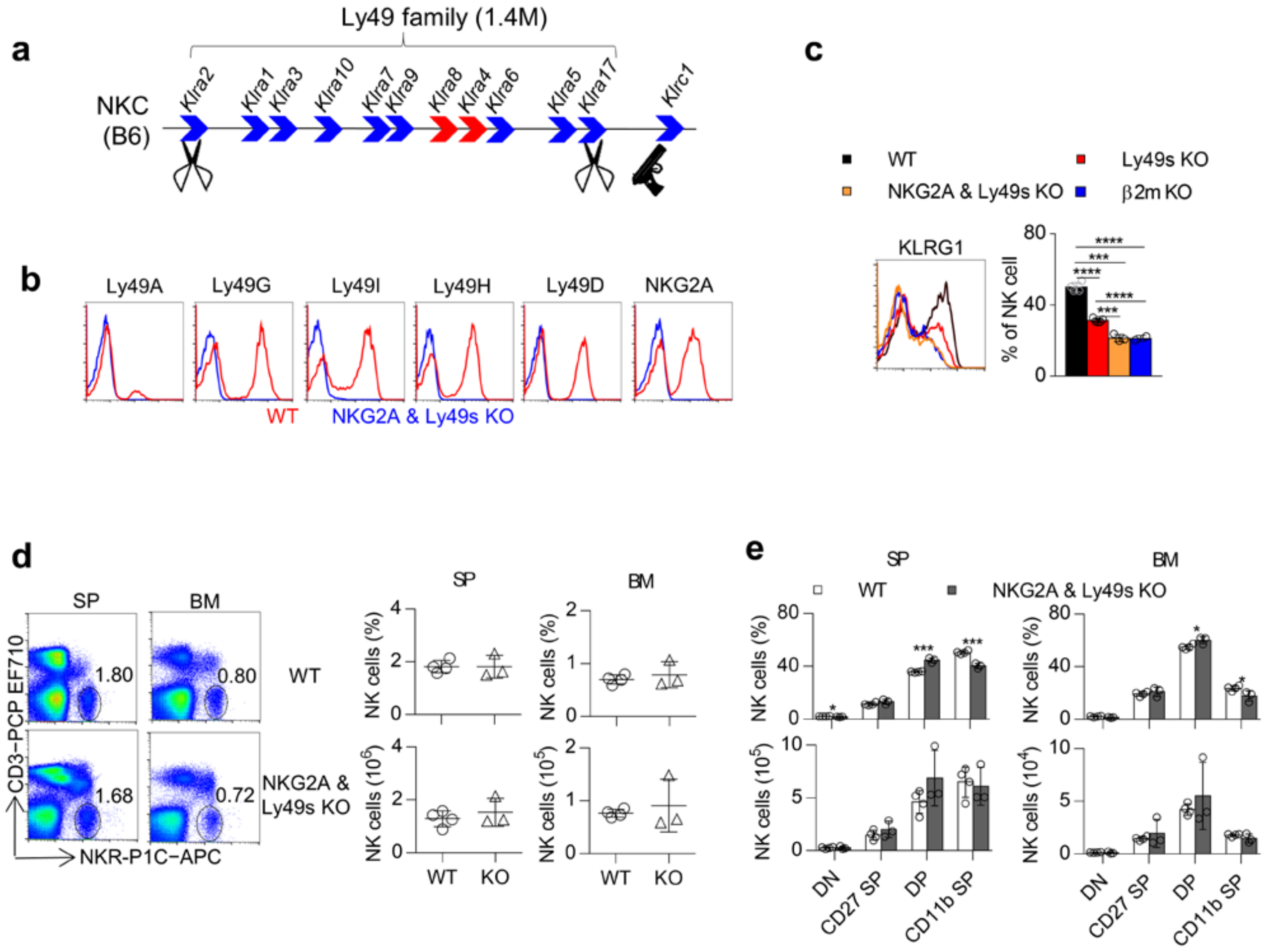

Supplementary Figure 7 | NK cell development in mice lacking Ly49 family and NKG2A. a. Diagram of the NKC locus. Guns and scissors denote CRISPR gRNAs targeting the indicated genes. **b.** Flow cytometry analysis of the expression of the indicated receptors on splenic $CD3^{-}NKp46^{+}$ NK cells from WT (red line) and NKG2A & Ly49s KO (blue line) mice. **c.** Representative flow cytometry histogram (left panel) and percentage (right panel) of KLRG1 expression on splenic NK cells ($CD3^{-}NKR\text{-}P1C^{+}$) from WT (black line) and indicated mutant mice. **d.** Representative flow cytometry plots (left panel), percentages and absolute numbers of gated $CD3^{-}NKR\text{-}P1C^{+}$ NK cells in the spleen (middle panel) and bone marrow (right panel) of WT and NKG2A & Ly49s KO mice. **e.** Analysis of NK cell development. Percentages (upper panel) and absolute numbers (lower panel) of gated $CD3^{-}NKR\text{-}P1C^{+}$ NK cells in the four stages of development, including DN ($CD27^{-}CD11b^{-}$), CD27 SP ($CD27^{+}CD11b^{-}$), DP ($CD27^{+}CD11b^{+}$), CD11b SP ($CD27^{-}CD11b^{+}$), in the spleen and bone marrow from WT and NKG2A & Ly49s KO mice. Each symbol represents an individual mouse. All data shown represent two independent experiments. Mean ± SD is shown. $*p < 0.05$, $***p < 0.001$ and $****p < 0.0001$. Unpaired Student's *t*-tests (two-tailed) was used to calculate these values. Source data are provided as a Source Data file.

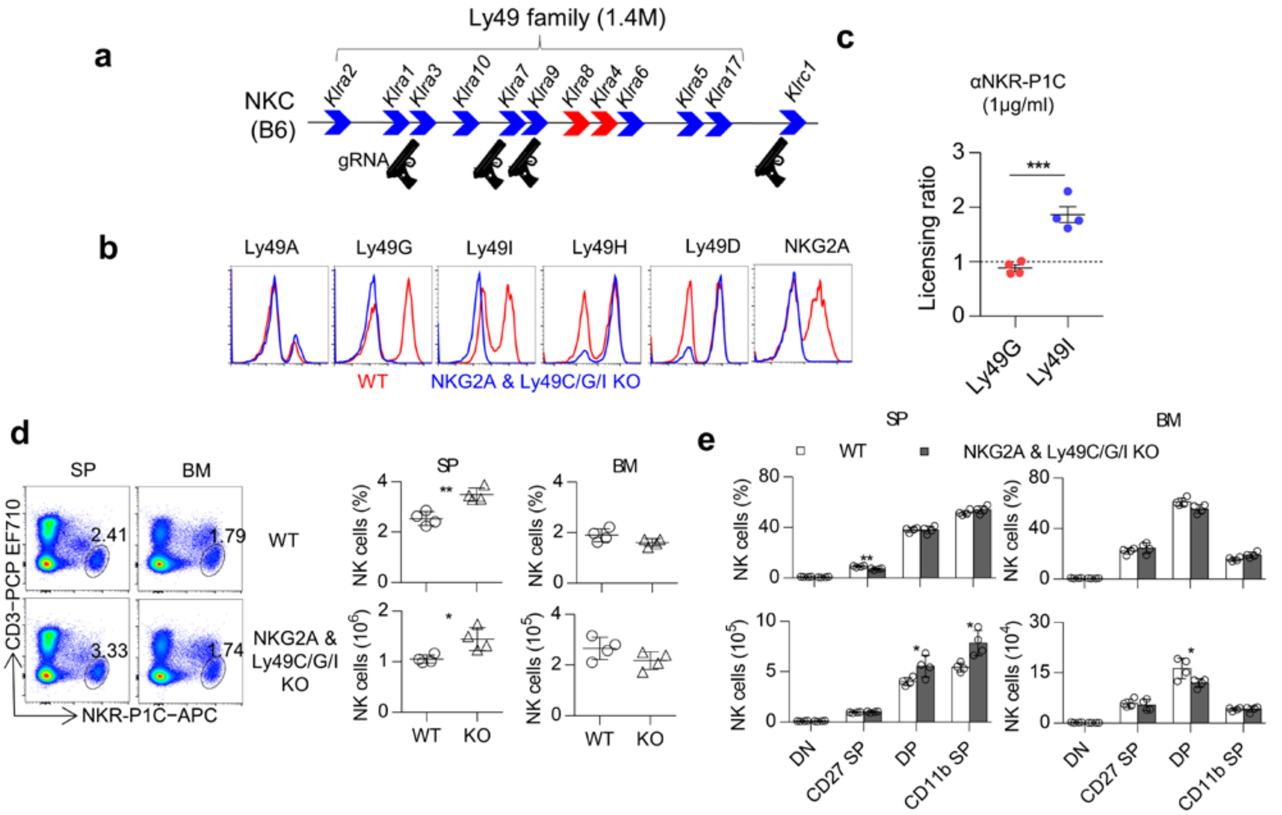

Supplementary Figure 8 | NK cell development in mice lacking inhibitory Ly49C/G/I and NKG2A. a. Diagram of the NKC locus. Guns denote CRISPR gRNAs targeting the indicated genes. **b.** Flow cytometry analysis (right panel) of expression of the indicated receptors on splenic $CD3^{-}NKp46^{+}$ NK cells from WT (red line) and NKG2A & Ly49C/G/I KO (blue line) mice. **c**. Licensing ratio of the indicated receptors for stimulation with plate-coated anti-NKR-P1C. Licensing ratio is relative production of IFN-γ by positive groups of indicated receptors comparing to negative groups of indicated receptors within the gated $CD3^{-}NKp46^{+}$ NK cells from the indicated mice. **d.** Representative flow cytometry plots (left panel), percentages and absolute numbers of gated $CD3^{-}NKR\text{-}P1C^{+}$ NK cells in the spleen (middle panel) and bone marrow (right panel) of WT and NKG2A & Ly49C/G/I KO mice. **e.** Analysis of NK cell development. Percentages (upper panel) and absolute numbers (lower panel) of gated $CD3^{-}NKR\text{-}P1C^{+}$ NK cells in the four stages of development, including DN ($CD27^{-}CD11b^{-}$), CD27 SP ($CD27^{+}CD11b^{-}$), DP ($CD27^{+}CD11b^{+}$), CD11b SP ($CD27^{-}CD11b^{+}$), in the spleen and bone marrow from WT and NKG2A & Ly49C/G/I KO mice. Each symbol represents an individual mouse. All data shown represent two independent experiments. Mean ± SD is shown. $^{*}p < 0.05$, $^{**}p < 0.01$ and $^{***}p < 0.001$. Unpaired Student's *t*-tests (two-tailed) was used to calculate these values. Source data are provided as a Source Data file.

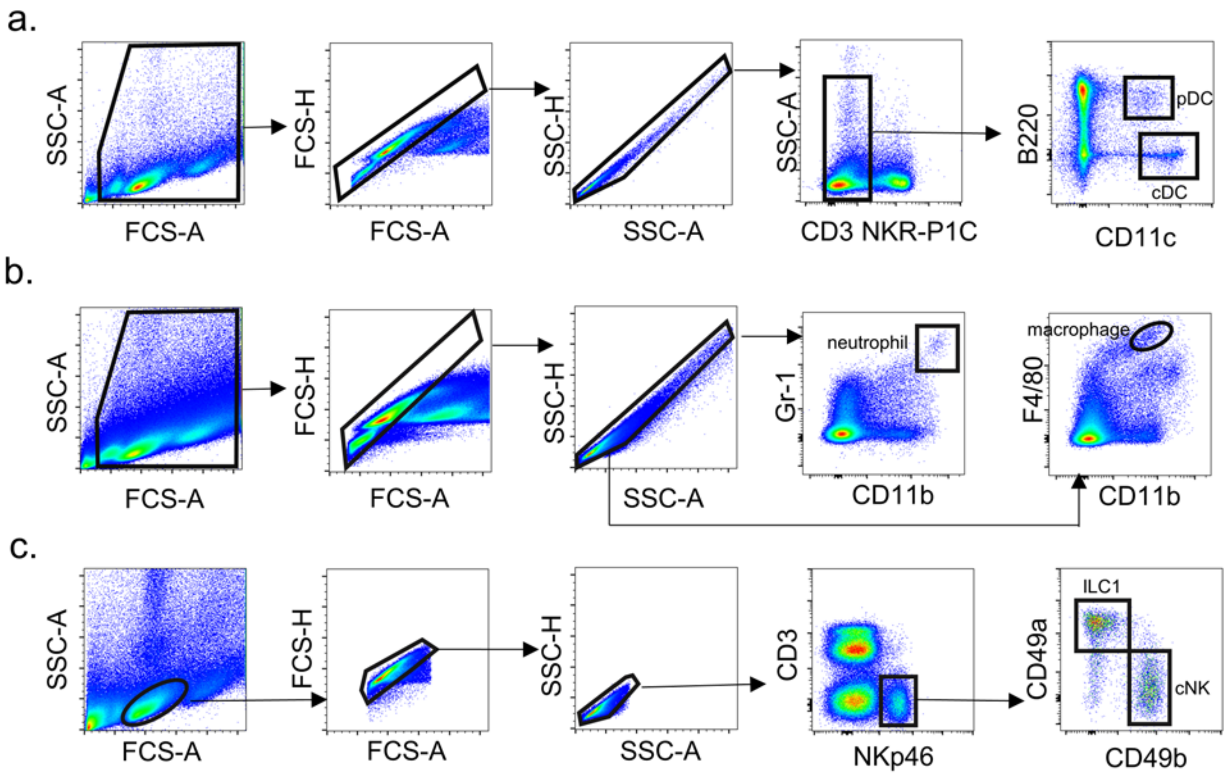

Supplementary Figure 9 | Gating strategies for the FACS data. (a-b). Gating strategies for supplementary Fig. 1d. **c.** Gating strategies for supplementary Fig. 1e.

Supplementary Table 1 | Primers for genomic PCR screening of Ly49-deficient mice

name	Forward primer	Reverse primer
P1	AACAAAGTGGTCGCAGTGTC	CTACAACCCAGGCCCCTTTA
P2	GAGCCACAATGTCTAGCTGC	TCATGACTGGTTGCTAGGCT
P3	TCCAGGAGTGAAGAAAAGGCA	TAAAAGTGCTGCAGGAGGGT
P4	GTGATCACCAAAGCCAGCAA	TGTGAGGAGAGGTGCATGTT
P5	AACTTGATCCCGTCTTCCCC	CCCAAAACACACACAAGCCA

Supplementary Table 2 | gRNAs for generating mutant mice

gene	gRNA No.	strand	Target (PAM)	
Klra2	#1	+	CCACGCGGCATTTGTCTTTTTGG	Intron1
	#2	−	GACAAATGCCGCGTGGTCTCTGG	Intron1
Klra17	#1	+	CCAGTAGTGACAATCGCGACAGG	After exon8
	#2	−	CTGAGGGGCGTAGAATAGGCGGG	After exon8
Klra3/9	#1	+	GTGAGACTTCATAAGTCTTCAGG	Exon2
	#2	+	TAAGGCATGAGGAGACTCAAGGG	Exon2
Klra4	#1	+	CCTGAAAAAGCTCGCCTCAGAGG	Exon2
	#2	+	CAGGGTTGCAGAACGAGATGAGG	Exon2
Klra8	#1	−	TGTTCAACCCTGAAGACTTGTGG	Exon2
	#2	+	AATCCTCTGTTCCCTTCGGCTGG	Exon3
Klrc1	#1	+	CTCCATTTCAGTCATCGAGCAGG	Exon1
	#2	+	TGCAGGAATTGCCCCTGCAAAGG	Exon1

Supplementary Table 3 | Primers for Real-time quantitative PCR

Primer name	Primer pair	Sequence	Product length
Klra1	Forward	CTATAGAGTGTGATCTTCTG	739 bp
	Reverse	CAAACAAATAAAACAGATTCGTCC	
Klra2	Forward	CCGTCAAGAGTACCAGGTCA	186 bp
	Reverse	CCTTCCACTTGCTTGCCTTT	
Klra3	Forward	TTGTAGGCCAAGCAATGAAACT	211 bp
	Reverse	AATGGGAACGCTATAATGCTGG	
Klra4	Forward	AAAAAGCTCGCCTCAGAGTTC	281 bp
	Reverse	AGTCCTGGTTTTATCACACAG	
Klra5	Forward	TATAGATTGCAGTCCAGGTGAG	492 bp
	Reverse	TTGGAAAGTTAACCAGGGAAATG	
Klra6	Forward	GCAGCTCATTGTGAAAGCTCT	218 bp
	Reverse	GAAGTTCATTGCCTGGCCTAG	
Klra7	Forward	TTGCCACGATAACTGCAGCCC	350 bp
	Reverse	AAATGTCTGAAGGAGCCAGGTT	
Klra8	Forward	ACAGTGAAACCAAGAGTGTTTC	473 bp
	Reverse	AGCTTTGTTTCTCTTCACAGCA	
Klra9	Forward	TGGAACAGTGAAACCAAGACG	158 bp
	Reverse	ATGGGAACGCTGTAATGCTG	
Klra10	Forward	AAGAGAACAGGACAGATGGAAT	167 bp
	Reverse	GGGAACGCTATAATGCTGGC	

Klra17	Forward	GTGCTCTATCCCCTGGCATC	157 bp
	Reverse	GGTGCTACAGTTATGGTGGCA	
Nkr-p1c	Forward	GAATCAGTGGGTGTGGGAAC	160 bp
	Reverse	GGTGGCTTTAAACCGAGGTAG	
Klrk1	Forward	TGCCATAATTACGACCTCAAGC	249 bp
	Reverse	GCACAATACTGGCTGAAACG	
