# Supplementary material for: Synergized regulation of NK cell education by NKG2A and specific Ly49 family members
Source: Nat Commun. 2019 Nov 1;10:5010. doi: 10.1038/s41467-019-13032-5 (PMC6825122; doi:10.1038/s41467-019-13032-5)
Supplement: Supplementary file 3 — Reporting Summary [file 41467_2019_13032_MOESM3_ESM.pdf]

nature research

Corresponding author(s): Zhongjun Dong

Last updated by author(s): Oct 15, 2019

Reporting Summary

Nature Research wishes to improve the reproducibility of the work that we publish. This form provides structure for consistency and transparency in reporting. For further information on Nature Research policies, see Authors & Referees and the Editorial Policy Checklist.

Statistics

For all statistical analyses, confirm that the following items are present in the figure legend, table legend, main text, or Methods section.

n/a	Confirmed	
☐	☒	The exact sample size (*n*) for each experimental group/condition, given as a discrete number and unit of measurement
☐	☒	A statement on whether measurements were taken from distinct samples or whether the same sample was measured repeatedly
☐	☒	The statistical test(s) used AND whether they are one- or two-sided<br>*Only common tests should be described solely by name; describe more complex techniques in the Methods section.*
☒	☐	A description of all covariates tested
☒	☐	A description of any assumptions or corrections, such as tests of normality and adjustment for multiple comparisons
☐	☒	A full description of the statistical parameters including central tendency (e.g. means) or other basic estimates (e.g. regression coefficient) AND variation (e.g. standard deviation) or associated estimates of uncertainty (e.g. confidence intervals)
☐	☒	For null hypothesis testing, the test statistic (e.g. *F*, *t*, *r*) with confidence intervals, effect sizes, degrees of freedom and *P* value noted<br>*Give P values as exact values whenever suitable.*
☒	☐	For Bayesian analysis, information on the choice of priors and Markov chain Monte Carlo settings
☒	☐	For hierarchical and complex designs, identification of the appropriate level for tests and full reporting of outcomes
☒	☐	Estimates of effect sizes (e.g. Cohen's *d*, Pearson's *r*), indicating how they were calculated

Our web collection on statistics for biologists contains articles on many of the points above.

Software and code

Policy information about availability of computer code

Data collection	BD FACSDiva (version 8.0)
Data analysis	Flow Jo (version 10.6.1), Prism7 for Windows (version 7.00)

For manuscripts utilizing custom algorithms or software that are central to the research but not yet described in published literature, software must be made available to editors/reviewers. We strongly encourage code deposition in a community repository (e.g. GitHub). See the Nature Research guidelines for submitting code & software for further information.

Data

Policy information about availability of data

All manuscripts must include a data availability statement. This statement should provide the following information, where applicable:

- Accession codes, unique identifiers, or web links for publicly available datasets
- A list of figures that have associated raw data
- A description of any restrictions on data availability

No datasets were generated or analysed during the current study.

Field-specific reporting

Please select the one below that is the best fit for your research. If you are not sure, read the appropriate sections before making your selection.

☒ Life sciences ☐ Behavioural & social sciences ☐ Ecological, evolutionary & environmental sciences

For a reference copy of the document with all sections, see nature.com/documents/nr-reporting-summary-flat.pdf

Life sciences study design

All studies must disclose on these points even when the disclosure is negative.

Sample size	For most in vitro and in vivo studies, sample sizes are more than 3 to overcome individual variation as in common practice.
Data exclusions	No data were excluded.
Replication	All major conclusions were supported by at least 2 independent experiments.
Randomization	Mice were randomly allocated into different groups.
Blinding	Investigators were blinded to group sets during data analysis.

Reporting for specific materials, systems and methods

We require information from authors about some types of materials, experimental systems and methods used in many studies. Here, indicate whether each material, system or method listed is relevant to your study. If you are not sure if a list item applies to your research, read the appropriate section before selecting a response.

Materials & experimental systems

n/a	Involved in the study	
☐	☒	Antibodies
☐	☒	Eukaryotic cell lines
☒	☐	Palaeontology
☐	☒	Animals and other organisms
☒	☐	Human research participants
☒	☐	Clinical data

Methods

n/a	Involved in the study	
☒	☐	ChIP-seq
☐	☒	Flow cytometry
☒	☐	MRI-based neuroimaging

Antibodies

Antibodies used	Monoclonal antibodies against mouse CD3 (#46-0033, clone eBio500A2, 1:200); B220 (#11-0452, clone RA3-6B2, 1:200); CD11c (#17-0114clone N418, 1:200); Gr-1 (#11-9668, 1A8-Ly6g, 1:200); F4/80 (#12-4801, clone BM8, 1:200); NKp46 (#11-3351, clone 29A1.4, 1:100); NKR-P1C (#17-5941, clone PK136, 1:200); CD49b (#12-5971, clone DX5, 1:200); CD122 (#12-1222, clone TM-b1, 1:200); CD117 (#48-1171, clone 2B8, 1:200); CD127 (#25-1273, clone SB/199, 1:200); Ly49A (#12-5856, clone A1, 1:200); Ly49I (#12-5895, clone YLI1-90, 1:200); Ly49H (#17-5886, clone 3D10, 1:200); Ly49G2 (#46-5781, clone eBio4D11, 1:200); NKG2D (#25-5882, clone CX5, 1:200); NKG2A (#46-5897, clone 16a11, 1:200); DNAM-1 (#17-2261, clone 10E5, 1:200); KLRG1 (#17-5893, clone 2F1, 1:200); CD11b (#17-0112, clone M1/70, 1:200); CD27 (#12-0271, clone LG.7F9, 1:200); MHC-I (#12-5999, clone 28-14-8, 1:200); CD45.1 (#48-0453, clone A20, 1:200); CD45.2 (#25-0454, clone 104, 1:200); IFN-γ (#12-7311, clone XMG1.2, 1:200) and CD107a (#50-1071, clone eBio1D4B, 1:100) were purchased from eBioscience. Monoclonal antibody against mouse CD49a (#74-0046, clone Ha31/8, 1:200); Ly49D (#555312, clone 4E5, 1:200) and Ly49C/I (#562055, clone 5E6, 1:200) were purchased from BD Biosciences.
Validation	All antibodies were validated by manufactures.

Eukaryotic cell lines

Policy information about cell lines

Cell line source(s)	MHC class I-deficient lymphoma cell line RMA-S cells (MHC-I deficient lymphomaoriginally generated by Klas Kärre lab, Karolinska Institute, Stockholm, Sweden and kindly provided by André Veillette, Clinical Research Institute of Montreal, Canada)). YAC-1 cells (thymoma, ATCC# TIB-160) and B16 F10 cells (melanoma, ATCC #CRL-6475) were purchased from ATCC.
Authentication	None of the cell lines used were authenticated.
Mycoplasma contamination	All cell lines tested for negative mycoplasma contamination.
Commonly misidentified lines (See ICLAC register)	N/A

Animals and other organisms

Policy information about studies involving animals; ARRIVE guidelines recommended for reporting animal research

Laboratory animals	All animals were described in the "Methods" section.
Wild animals	N/A
Field-collected samples	N/A
Ethics oversight	All experiments using mice were approved by the Animal Ethics Committee of Tsinghua University.

Note that full information on the approval of the study protocol must also be provided in the manuscript.

Flow Cytometry

Plots

Confirm that:

☒ The axis labels state the marker and fluorochrome used (e.g. CD4-FITC).

☒ The axis scales are clearly visible. Include numbers along axes only for bottom left plot of group (a 'group' is an analysis of identical markers).

☒ All plots are contour plots with outliers or pseudocolor plots.

☒ A numerical value for number of cells or percentage (with statistics) is provided.

Methodology

Sample preparation	Sample preparation was described in "Methods" section.
Instrument	BD LSR II (four-laserBlue/Red/Violet/ultraviolet flow cytometry analyzer, BD Biosciences)
Software	BD FACSDiva (version 8.0), Flow Jo (version 10.6.1), Prism7 for Windows (version 7.00)
Cell population abundance	N/A
Gating strategy	Gating strategies were described in supplementary Figure 9.

☒ Tick this box to confirm that a figure exemplifying the gating strategy is provided in the Supplementary Information.
